# Integrated metabolomics and transcriptomics study of traditional herb *Astragalus membranaceus Bge. var. mongolicus (Bge.) Hsiao* reveals global metabolic profile and novel phytochemical ingredients

**DOI:** 10.1186/s12864-020-07005-y

**Published:** 2020-11-18

**Authors:** Xueting Wu, Xuetong Li, Wei Wang, Yuanhong Shan, Cuiting Wang, Mulan Zhu, Qiong La, Yang Zhong, Ye Xu, Peng Nan, Xuan Li

**Affiliations:** 1 Key Laboratory of Synthetic Biology, CAS Center for Excellence in Molecular Plant Sciences, Institute of Plant Physiology and Ecology, Chinese Academy of Sciences, Shanghai, 200032 China; 2 University of Chinese Academy of Sciences, Beijing, 100049 China; 3 Ministry of Education Key Laboratory for Biodiversity Science and Ecological Engineering, School of Life Sciences, Fudan University, Shanghai, 200438 China; 4 Shanghai Chenshan Plant Science Research Center, Shanghai Chenshan Botanical Garden, Shanghai, 201602 China; 5 Research Institute of Biodiversity & Geobiology, Department of Life Science, Tibet University, Lhasa, China 850000 China; 6 Department of Colorectal Surgery, Fudan University Shanghai Cancer Center, Shanghai, China

**Keywords:** *Astragalus membranaceus Bge. Var. mongolicus*, Non-targeted metabolomics, Phytochemical compositions, Secondary metabolites, Flavonoid derivative, Biosynthesis

## Abstract

**Background:**

*Astragalus membranaceus Bge. var. mongolicus (Bge.) Hsiao* is one of the most common herbs widely used in South and East Asia, to enhance people’s health and reinforce vital energy. Despite its prevalence, however, the knowledge about phytochemical compositions and metabolite biosynthesis in *Astragalus membranaceus Bge. var. mongolicus (Bge.) Hsiao* is very limited.

**Results:**

An integrated metabolomics and transcriptomics analysis using state-of-the-art UPLC-Q-Orbitrap mass spectrometer and advanced bioinformatics pipeline were conducted to study global metabolic profiles and phytochemical ingredients/biosynthesis in *Astragalus membranaceus Bge. var. mongolicus (Bge.) Hsiao*. A total of 5435 metabolites were detected, from which 2190 were annotated, representing an order of magnitude increase over previously known. Metabolic profiling of *Astragalus membranaceus Bge. var. mongolicus (Bge.) Hsiao* tissues found contents and synthetic enzymes for phytochemicals were significantly higher in leaf and stem in general, whereas the contents of the main bioactive ingredients were significantly enriched in root, underlying the value of root in herbal remedies. Using integrated metabolomics and transcriptomics data, we illustrated the complete pathways of phenylpropanoid biosynthesis, flavonoid biosynthesis, and isoflavonoid biosynthesis, in which some were first reported in the herb. More importantly, we discovered novel flavonoid derivatives using informatics method for neutral loss scan, in addition to inferring their likely synthesis pathways in *Astragalus membranaceus Bge. var. mongolicus (Bge.) Hsiao*.

**Conclusions:**

The current study represents the most comprehensive metabolomics and transcriptomics analysis on traditional herb *Astragalus membranaceus Bge. var. mongolicus (Bge.) Hsiao*. We demonstrated our integrated metabolomics and transcriptomics approach offers great potentials in discovering novel metabolite structure and associated synthesis pathways. This study provides novel insights into the phytochemical ingredients, metabolite biosynthesis, and complex metabolic network in herbs, highlighting the rich natural resource and nutritional value of traditional herbal plants.

## Background

As a member of the legume family, *Astragalus membranaceus* Bge. var. *mongolicus* (Bge.) Hsiao (AMM for short) is a perennial herbaceous plant grown in northwestern China, having a plant-to-harvest cycle of 2 to 3 years [1, 2]. AMM is one of the common herbs that is widely used in the South and East Asia, to enhance people’s health and reinforce vital energy. The dried root of AMM, the main herbal material, was reported to have anti-perspirant, anti-diuretic, antitumor, anti-oxidation, and anti-inflammation effect [1, 3–6]. The major bioactive components of AMM are a variety of flavonoids, terpenoids, polysaccharides, amino acids, etc. Up-to date, about 150 different metabolites were identified from AMM, including saponins, flavonoids, polysaccharides, and amino acids [7]. Calycosin, calycosin-7-O-b-D-glucoside (CG), and astragaloside IV (ASI) are considered major bioactive constituents from AMM [8–10]. Calycosin and CG are isoflavones that were reported to have anti-inflammatory, anti-radiation, anti-cancer, and anti-microbial activities, and act as antioxidant and adjuvant agents [11–13]. The effect of CG was also reported as a hyaluronidase inhibitory component that was used in anti-osteoarthritis treatment [12, 13]. ASI, a natural triterpenoid saponins from AMM, was reported to have effects of anti-fatigue, anti-cancer, anti-coxsackie B virus, etc. [10, 14]. Indeed, these ingredients were used as standard compositions to evaluate the quality of AMM harvests.

Despite being one of the most important herbs, knowledge about AMM phytochemical compositions and metabolite biosynthesis is very limited. Metabolomics seeks to provide a comprehensive profile of all metabolites present in a biological sample under certain conditions [15, 16]. With the advance of mass spectrum (MS) technology, plant metabolomics presents a new perspective to understand the chemical compositions and functions of the traditional herb like AMM. In the past decades, the study of metabolomics of AMM was based on targeted analysis [17, 18] focusing on some of the active ingredients of AMM, including astragalosides, flavones, and polysaccharides. Recently, liquid chromatography-mass spectrum (LC-MS) has been widely applied in metabolomics studies, with technical advance in wider range of separation, higher selectivity, and higher sensitivity [19]. Metabolomics analyses using advanced LC-MS systems were performed on a number of plants, including Arabidopsis [20, 21], *Oryza sativa* [22, 23], Tomato [24, 25], which was proven effective for metabolite identification and pathway elucidation in plants [26, 27]. In metabolomics analysis, tandem MS generates informative fragment peaks, forming fingerprints specific to the detected molecules [28]. Annotation of detected molecules from their fingerprints has been a major challenge for MS studies, because of the limited data from reference mass spectra. Recently, computational approach to simulate compound fragmentation was developed for annotating MS data. A number of in silico tools, like MetFrag [28], FingerID [29], CFM-ID [30], and MetFusion [31], were launched to allow users to query broader chemical structure databases, like KEGG, PubChem, etc. [32].

A comprehensive metabolomics and transcriptomics analysis using advanced LC-MS combined with transcriptome analysis is desired to study AMM and understand their chemical compositions and metabolite biosynthesis in vivo. In current study, we performed a non-targeted metabolomics analysis on three AMM tissues: leaf, root, and stem. An unprecedented number of stable metabolites (5435) were discovered in AMM, and 2190 metabolites were annotated by our integrated bioinformatics pipeline*.* Metabolic profiling found that metabolites have different accumulation patterns in AMM tissues. We also performed transcriptome analysis to explore the mechanism underlying the metabolic regulation among three tissues in AMM. The three tissues also presented different expression patterns, and pointed to importance of all the tissues for the production of bioactive compounds. Furthermore, we inferred the complete pathways of Phenylpropanoid, Flavonoid, and Isoflavonoid biosynthesis in AMM by integrating its metabolomics and transcriptomics data, including critical enzymes and metabolites. We further discovered novel derivatives for secondary metabolites, like isoflavonoids, from modifications with various chemical groups. This study reveals the comprehensive profile of metabolic activities on herb AMM, providing novel insights into phytochemical ingredients and metabolite biosynthesis in traditional herbs.

## Results and discussion

### Metabolomics analysis revealing abundant metabolites in AMM tissues using an integrated informatics pipeline

While root is the main herbal material from AMM, it was found that secondary metabolites was produced in various AMM tissues [2, 33]. We carried out a deep metabolomics study on the different tissues of AMM, i.e. leaf, root and stem, using non-targeted MS-analysis protocol with state-of-art UPLC-Q-Orbitrap mass spectrometer and an integrated informatics pipeline [34] (*Methods*, Additional file 1). Mass spectral data were processed by alignment of all data-sets from the AMM tissues (each with three biological replicates) and controls (*Methods*). After removing redundancy and noise signals, which are of poor quality or non-biological origin [35], 11,101 and 9250 spectra signals were retained for positive and negative mode respectively. The spectra signals for positive and negative mode were merged to obtain a total of 5435 significant peaks from AMM tissues, for which 3985 were tagged with MS2 spectrum (Additional file 2)*.*

Annotation of metabolites with MS2 tags from AMM was performed by comparison of the accurate m/z values and the fragmentation patterns based on the metabolites above [36]. We annotated the metabolites by matching their spectra data against experimental MS reference databases (similarity score threshold 0.8; *Methods*). Those do not have any match were subsequently annotated with virtual MS reference databases. Taken together, 2190 metabolites from AMM were annotated by our integrated bioinformatics pipeline (Additional file 3). Among them, the most abundant are Terpenoids (254), followed by Flavonoids (101), Alkaloids (97), Phenylpropanoids (57), and Fatty acids related compounds (35) (Table 1). We found an unprecedented number of metabolites from AMM, for which we have increased the number of annotated metabolites by an order of magnitude over previous studies. Note many metabolites previously identified from AMM were included in our list (Additional file 4).

**Table 1:** Classification of annotated metabolites from AMM

category	counts
Terpenoids	254
Flavonoids	101
Alkaloids	97
Phenylpropanoids	57
Fatty acids related compounds	35
Skimate / acetate-malonate pathway derived compounds	11
Polyketides	10
Amino acid related compounds	4

To understand the metabolic activities in AMM, we mapped the annotated metabolites to KEGG pathways. 1330 metabolites were mapped to KEGG and assigned to 247 reference canonical KEGG pathways, which including 144 metabolites in the pathway of ‘Biosynthesis of secondary metabolites’ (Additional file 5). These metabolites cover most of central metabolism and reflect the physiological state and the edible and nutritive value of AMM.

### Transcriptomics analysis of gene expression in AMM tissues

**Fig. 1:**
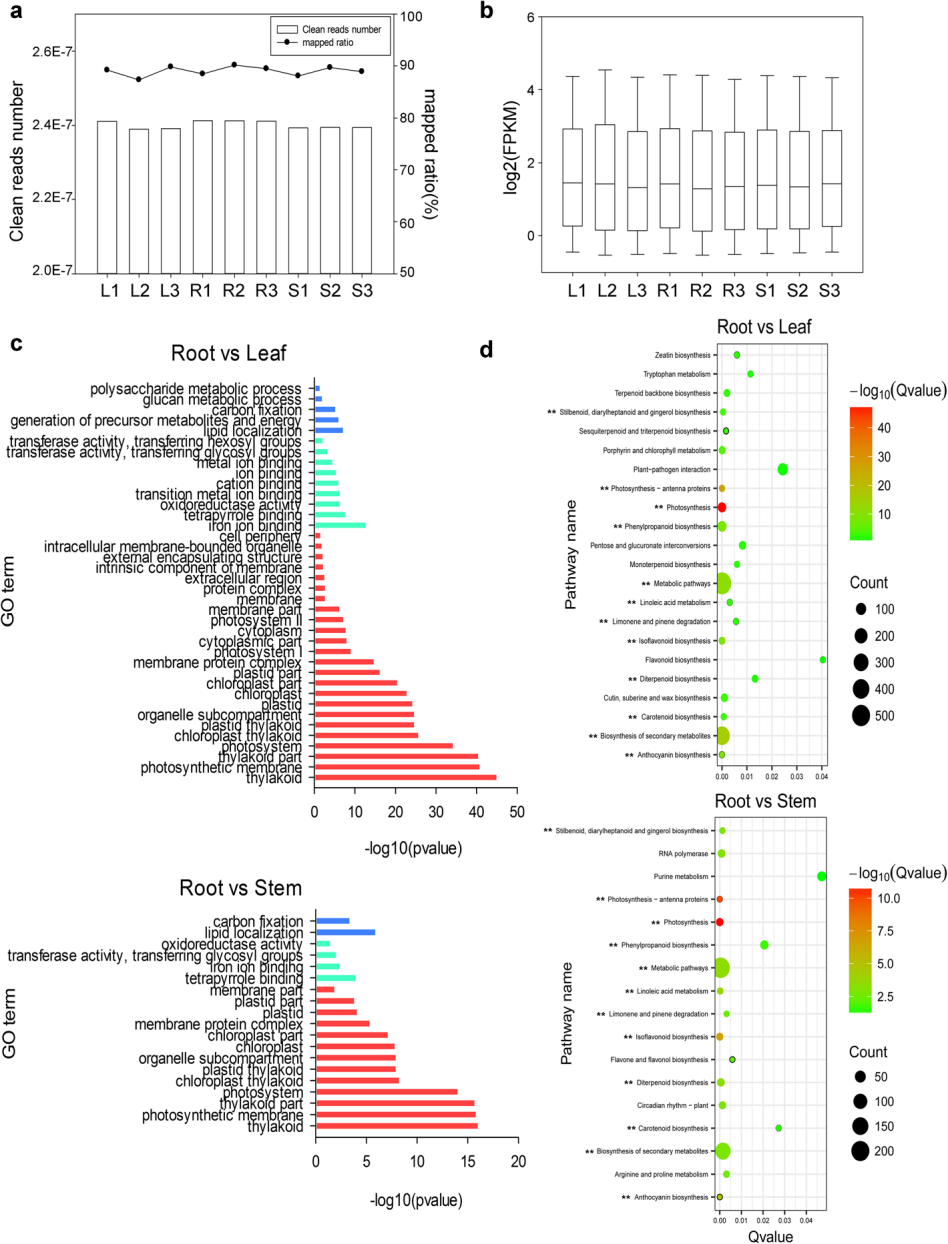
Functional annotation and classification of differentially expressed unigenes between different tissues of AMM. **a** The reads number and mapped ratio to the reference transcriptome of different tissues (L1-L3:leaf; R1-R3:root; S1-S3: stem). The bar plot represents the clean reads number in different tissues, and the dots plot represents mapped ratio of different tissues to the reference transcriptome in different tissues. **b** The expression level of unigenes in different tissues from AMM (FPKM ≥0.5). We took the logarithm of each FPKM value of all unigenes. **c** Gene Ontology (GO) functional classifications of differentially expressed unigenes (Only list significantly enriched GO terms, *P* value≤0.05). Red: CC Cellular Component; Green: MF Molecular Function; Blue: BP Biological Process. **d** Significantly enriched KEGG pathways between differentially expressed unigenes. The Q value denoted the corrected *P*-value (Significant pathways were identified by Q value≤0.05). Count denoted the number of differentially expressed unigenes mapped to a certain pathway according to KEGG database. The pathways with ‘**’ denoted the significantly enriched pathways that included in both between the root vs leaf and root vs stem analysis

Transcriptomics analysis was conducted by RNA-seq on AMM tissues, leaf, stem, and root with three replicates for each tissue (Additional file 1). A total of 3.60, 3.62 and 3.59 Gb RNA-seq data were generated for leaf, stem, and root, respectively (Fig. 1a). Clean reads were mapped to reference transcriptome [2] with an average mapping ratio ~ 89.0%, validating that our RNA-seq data has high sequencing precision. There were on average 65,000 unigenes expressed (FPKM ≥0.5) in three AMM tissues. Although each tissue had similar distribution of gene expression levels, denoted by their FPKM values (Fig. 1b), we found leaf and stem had differentially expressed genes from root, particularly in some secondary metabolic pathways, which points to important roles of AMM leaf and stem in production of bioactive compounds.

Compared to root transcriptome, there were 3629, and 1358, unigenes differentially expressed in leaf and stem, respectively (Additional file 6, Additional file 7A and B). To understand their functions in leaf and stem, GO analysis were performed to assign them to biological process, cell component, or molecular function. Compared to root, the most significant differences in leaf and stem were related to chloroplast/thylakoid structure and functions (Fig. 1c). Again, KEGG analysis indicated leaf and stem have significantly altered activities in the biosynthesis pathways of many secondary metabolites, like isoflavonoids and terpenoids (Fig. 1d).

### Profiling metabolic activities of AMM tissues by integrated metabolomics and transcriptomics analysis

The metabolic activities of AMM tissues were analyzed by combining metabolomics and transcriptomics data. First, the levels of the 5435 metabolic features in AMM were defined for leaf, root and stem. Surprisingly we found leaf had the most abundant metabolites, whereas root had the least (Additional file 8A). This was consistent with the study that showed leaf had the highest number of expressed unigenes in many metabolic pathways [2]. The relative intensity of the metabolites displayed similar pattern, in which root had significant lower quantities of metabolites than either leaf or stem (Mann-Whitney Rank Sum Test, *P* < 0.001) (Additional file 8A).

Principal Component Analysis (PCA) was carried out as an unsupervised analysis on the metabolomic profiles for AMM tissues (*Methods*). The three AMM tissues were well separated by the first and second major components, for which 46.1 and 21.9% of the variance were explained by the first two main principal components, respectively (Additional file 8B). The result indicated systematic difference in metabolites among the AMM tissues. To understand the major differences in metabolite levels between leaf and root, and stem and root, we then conducted orthogonal partial least squares discriminate analysis (OPLS-DA) [37] and determined the featured metabolites for them. For differences between leaf and root, the tissue samples were well separated in the model (Additional file 8C). The *R*^2^*X*, *R*^2^*Y* (goodness-of-fit parameter) and *Q*^2^ (predictive ability parameter) of the OPLS-DA model are 0.814, 1, and 0.995, respectively, indicating good quality and high confidence of our model. S-plot was used to find the featured metabolites distinguishing leaf and root (Additional file 8D). We then performed VIP (variable importance in the projection) prediction with a permutation test (*n* = 200) to validate the models’ reliability (Additional file 8E), and obtained 2020 significantly differential metabolites (Additional file 7C) with threshold VIP value > 1. The differential metabolites between leaf and root included Chikusetsusaponin V, Astragaloside IV, Isoastragaloside II, Formononetin, etc. Similar analysis was also carried out between stem and root (Additional file 9, 2,050 significantly differential metabolites, Additional file 7D). The differential metabolites between stem and root included Chikusetsusaponin V, Astragaloside IV, Quillaic acid, Soyasapogenol B, Oleanolic acid, etc.

### Phenylpropanoids

**Fig. 2:**
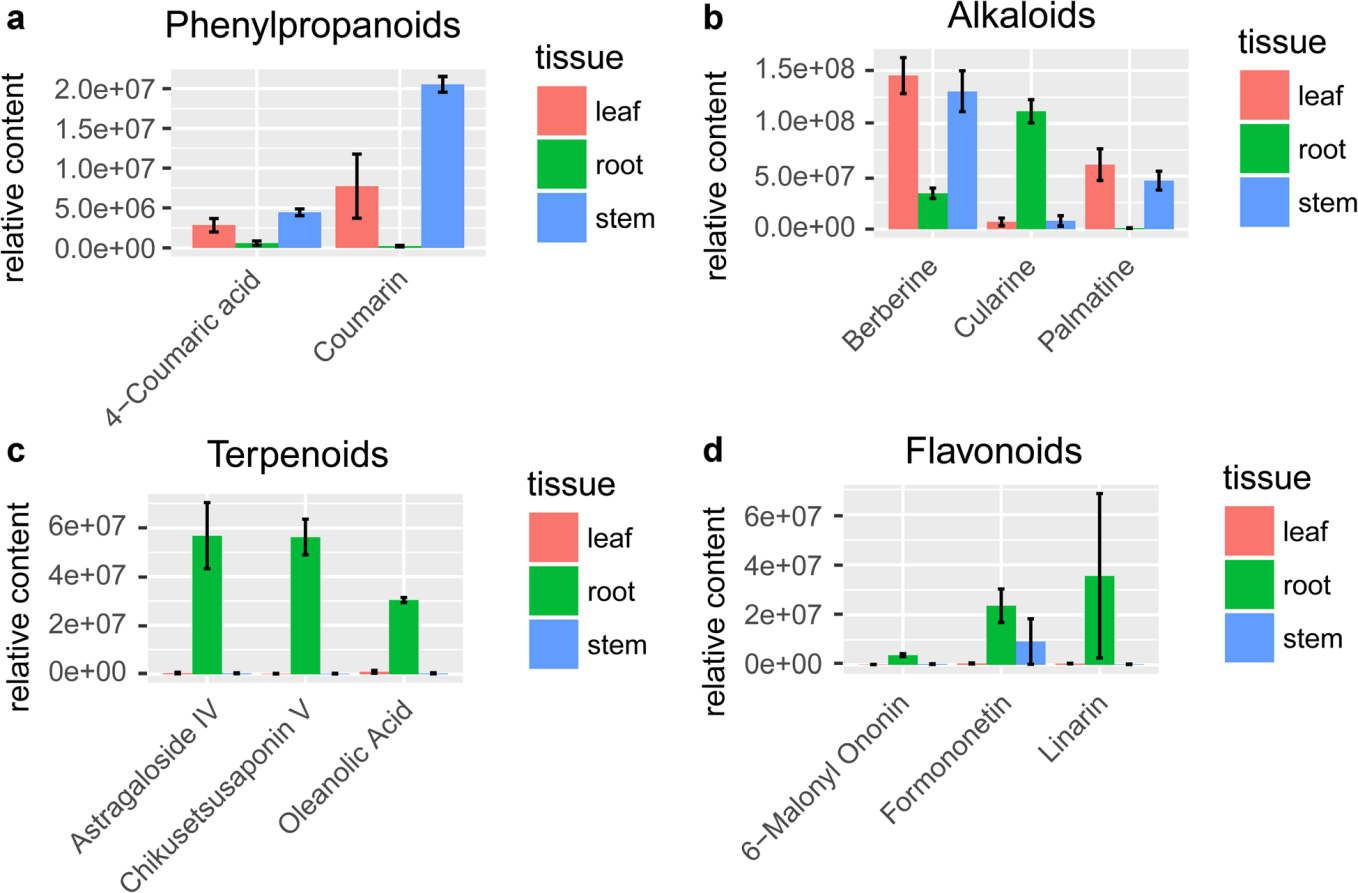
The relative content (GroupArea) of metabolites. **a** phenylpropanoids, **b** alkaloids, **c** terpenoids, and **d** flavonoids in different tissues of AMM

The phenylpropanoid biosynthesis is upstream of the flavonoid biosynthesis pathway [38]. There are 57 metabolites from AMM that were annotated as phenylpropanoids (Additional file 3). In general, the average content of phenylpropanoid in the root was significantly lower than in the stem (Mann-Whitney Rank Sum Test *P* < 0.001 (root vs stem)), meanwhile the average content of phenylpropanoid in the root was significantly lower than in the leaf, although statistical significance was not reached (Additional file 10A). Among these phenylpropanoid metabolites, 22 were mapped to the phenylpropanoid biosynthesis pathway. In phenylpropanoid biosynthesis pathway, Coumaric acid, sinapic acid, and cinnamic acid, are precursors for coumarin synthesis [39]. Coumarins are a large family of naturally occurring substances of fused benzene and a-pyrone rings that were found primarily in popular medicines [40]. Coumarins are regarded as phytoalexins [41], which are notable for their role as anticancer, anti-inflammatory, antimicrobial, anti-oxidant and anticoagulant bioactive compounds [42–44]. The contents of cinnamic acid and 4-Coumaric acid were much more abundant in the leaf and stem than that in the root of AMM (Fig. 2a). Cinnamic acid is transformed into Cinnamoyl-CoA and coumaryol-CoA, which are direct precursors for flavonoid biosynthesis.

### Alkaloids

There are 97 alkaloids annotated for AMM (Additional file 3). These alkaloids account for different types of alkaloids: Isoquinoline alkaloids (21), Amines (14), Pyridine alkaloids (7), Piperidine alkaloids (11), etc. They were mapped to KEGG pathways, including biosynthesis of alkaloids derived from shikimate pathway (19), Tropane, piperidine and pyridine alkaloid biosynthesis (18), isoquinoline alkaloid biosynthesis (16), etc. In general, the relative contents of alkaloids in root were lower than the other two tissues, leaf or stem (Additional file 10B), although statistical significance was not reached. Despite, the relative content of Cularine in the root was 16.73 and 33.96 times the amount of that in the leaf and stem, respectively (Fig. 2b). Cularine was reported to have non-specific antispasmogenic activity on guinea-pig and human airways [45]. On the other hand, for Palmatine, a protoberberine alkaloids (isoquinoline alkaloid), the relative content in the leaf and stem were 79.37 and 45.24 times the amount of that in the root (Fig. 2b). Palmatine exerts a wide range of functions in vivo, with sedative effect, broad-spectrum antibacterial property, antioxidant activities [46], immunity enhancement, and relief of diabetic neuropathic pain and depression [46–48]. Another protoberberine alkaloids, Berberine, which also had a significantly higher relative content in the leaf than in the root and stem (Fig. 2b), demonstrated efficacy in anti-diabetic, anti-inflammatory, anti-dyslipidemia [49], and antioxidant activities [46]. These results suggested that other tissues besides AMM root may have potential nutritive value in functions like blood circulation, glucose regulation, and immunity enhancement [50].

### Terpenoids

There are 254 terpenoids annotated for AMM in our study. They consisted of 12 monoterpenoids, 57 sesquiterpenoids, 81 diterpenoids, and 104 triterpenoids. They were mapped mainly to the monoterpenoid biosynthesis, diterpenoid biosynthesis, sesquiterpenoid and triterpenoid biosynthesis, and biosynthesis of terpenoids and steroids pathways. Their average content in the root was significantly lower than in leaf or stem (Mann-Whitney Rank Sum Test: *p* < 0.001 (root vs leaf); p < 0.001 (root vs stem)) (Additional file 10C). These results agree with the previous study that leaf had a higher content of a few terpenoids (saponins) than the root [50]. Our analysis further identified that at least 23 terpenoids accumulated significantly higher in root than in leaf or stem (VIP > 1 and fold change ≥2), respectively.

Astragaloside IV (AMM5250) was known as the main active components of AMM and plays a role in anti-inflammatory, antioxidant, regulating energy metabolism, protectionnervous, anti-cancer [51], treating diabetes, etc. [52]. Astragaloside IV in the root, representing the highest content among differential terpenoids, was 219.49 and 194.77 times the amount of that in leaf and stem, respectively (Fig. 2c). Chikusetsusaponin V (AMM5423) in the root, not reported in AMM, was the second highest content among differential terpenoids, which was 343.74 and 308.81 times the amount of that in leaf and stem, respectively (Fig. 2c). Chikusetsusaponin V was reported to exhibit neuroprotective function [53], inhibit inflammatory responses, and reduce blood lipid [54, 55]. In addition, Oleanolic acid (AMM4067) in root was 32.90 and 205.79 times the amount of that in leaf and stem, respectively (Fig. 2c). Oleanolic acid was widely used for treating hepatopathy, as it can protect the liver from liver damage [56]. It is also an ingredient in skin care products to repair damaged cells and promote cell regeneration [56]. Oleanolic acid and its derivatives were also suggested to have bioactivities of antiosteoporosis, antidiabetes, antibacterial, anticancer and hemolytic effects [56]. The triterpenoids in AMM root were found to mostly belong to Oleananes and Protostanes, which we found to have mainly hexose group for glycosylation modification. Oleananes and Protostanes were reported to have function in regulating lymphocyte proliferation [57], in inflammatory response [58], and anticancer activities [58, 59].

### Terpenoids biosynthetic pathway

**Fig. 3:**
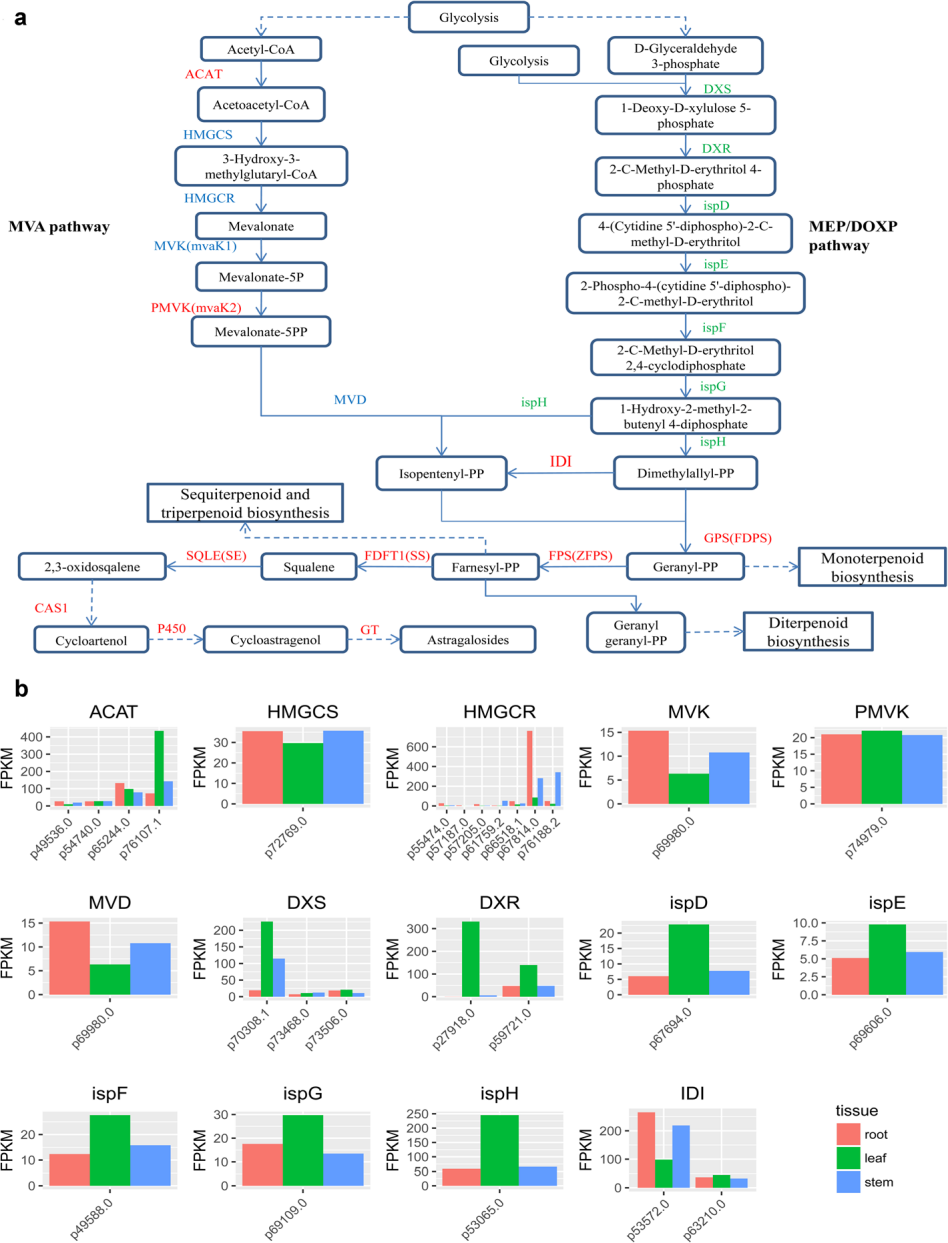
The inferred pathways for Terpenoids biosynthesis in Mongolicus. **a** The Terpenoids biosynthesis pathway. The enzymes confirmed by transcriptomics data are shown in red. Blue indicate the highly expressed enzymes in root and stem, while green indicate the highly expressed in leaf. ACAT: acetyl-CoA C-acetyltransferase; HMGCS: hydroxymethylglutaryl-CoA synthase; HMGCR: hydroxymethylglutaryl-CoA reductase; MVK: mevalonate kinase; PMVK: phosphomevalonate kinase; MVD: diphosphomevalonate decarboxylase; DXS: 1-deoxy-D-xylulose-5-phosphate synthase;DXR:1-deoxy-D-xylulose-5-phosphate reductoisomerase; ispD:2-C-methyl-D-erythritol 4-phosphate cytidylyltransferase; ispE: 4-diphosphocytidyl-2-C-methyl-D-erythritol kinase; ispF: 2-C-methyl-D-erythritol 2,4-cyclodiphosphate synthase; ispG:(E)-4-hydroxy-3-methylbut-2-enyl-diphosphate synthase; ispH: 4-hydroxy-3-methylbut-2-en-1-yl diphosphatereductase; IDI: isopentenyl-diphosphate Delta-isomerase. FDPS:farnesyldiphosphate synthase; ZFPS:(2Z,6Z)-farnesyldiphosphate synthase; FDFT1: farnesyl-diphosphatefarnesyltransferase; SQLE: squalenemonooxygenase. CAS1: cycloartenol synthase; P450: cytochromeP450; GT: glycosyltransferase. **b** The expression of the genes (enzymes) in the pathway measured by FPKM value

In higher plants, the biosynthesis of triterpenoid saponins remains unsolved. The terpenoid backbone biosynthesis pathway was the base of the biosynthesis of triterpenoid saponins. Hence, we first investigated the AMM genes involved in the terpenoid backbone biosynthesis pathway (Fig. 3a), and found all the predicted enzymes involved in the pathway. Additionally, we found different patterns of MVA and MEP/DOXP pathway genes in AMM tissues. While four enzymes in the MVA pathway were highly expressed in root and stem, eight in the MEP/DOXP pathway were highly expressed in leaf, consistent with the action of non-mevalonate (MEP/DOXP) pathway in chloroplasts (Fig. 3b) [2].

We found some high levels of astragalosides in root and stem, where MVA pathway was dominant. Downstream the terpenoid backbone biosynthesis, astragalosides (triterpenoid) were synthesized via the sesquiterpenoid and triterpenoid biosynthesis pathway (Fig. 3a). The sesquiterpenoid and triterpenoid biosynthesis pathway includes cycloartenol synthase (CAS1), cytochromeP450 (P450), and glycosyl transferase(GT). We discovered four isoforms for CAS1, p48468.0, p62650.0, p69652.1 and p75841.1, for which three are new ones. Among these, p48468.0 and p62650.0 were expressed specifically in root. p69652.1 was highly expressed in root and stem, whereas p75841.1 was similarly expressed in three tissues. On other hand, the content of ~ 20 monoterpenoids and diterpenoids are higher in leaf than in root and stem (VIP > 1 and fold change ≥2). This suggested that monoterpenoids and diterpenoids are synthesized through the MEP/DOXP pathway, which is dominant in leaf.

### Flavonoids

Flavonoids, especially its subclass isoflavonoids, were suggested to possess antibacterial and antioxidant functions [2]. They have a wide range of clinical usage, e.g. prevention of cancer and neurodegenerative diseases [60]. There are 101 flavonoids annotated for AMM, including flavones, isoflavone, flavanones, chalcones, isoflavanones, flavonols, and anthocyanins. We found glycosylation is the most frequent modification in flavonoids, and the glycosylation groups are mainly glucoside, and rhamnoside. The average content of flavonoids in root, in general, was significantly lower than in leaf and stem (Mann-Whitney Rank Sum Test: *p* < 0.001 (root vs leaf), *p* < 0.001 (root vs stem)) (Additional file 10D).

Despite the general lower contents of flavonoid in the root, we found five flavonoids accumulated significantly higher in root (VIP > 1 and fold change ≥2) than in leaf and stem. Formononetin [61] had the highest relative content in differential flavonoid metabolites, and was more abundant in root, which has 39.09 and 65.49 times the amount of that in leaf and stem, respectively (Fig. 2d). Formononetin was reported to have anti-angiogenic effect in treatment of colon cancer cells in vitro and in vivo [62]. Formononetin 7-O-glucoside-6″-O-malonate (6-malonyl ononin), a derivative of formononetin, in root was 24.24 and 8.98 times the amount of that in leaf and stem (Fig. 2d). Linarin (AMM4734) in root was 12.52 and 23.84 times the amount of that in leaf and stem, respectively (Fig. 2d). It was reported to have functions of anti-aging, anti-hypoxia, inhibition of hyperglycemia, anti-acetylcholinesterase and neuroprotective [63, 64].

### Phenylpropanoid, flavonoid, and Isoflavonoid biosynthetic pathways

**Fig. 4:**
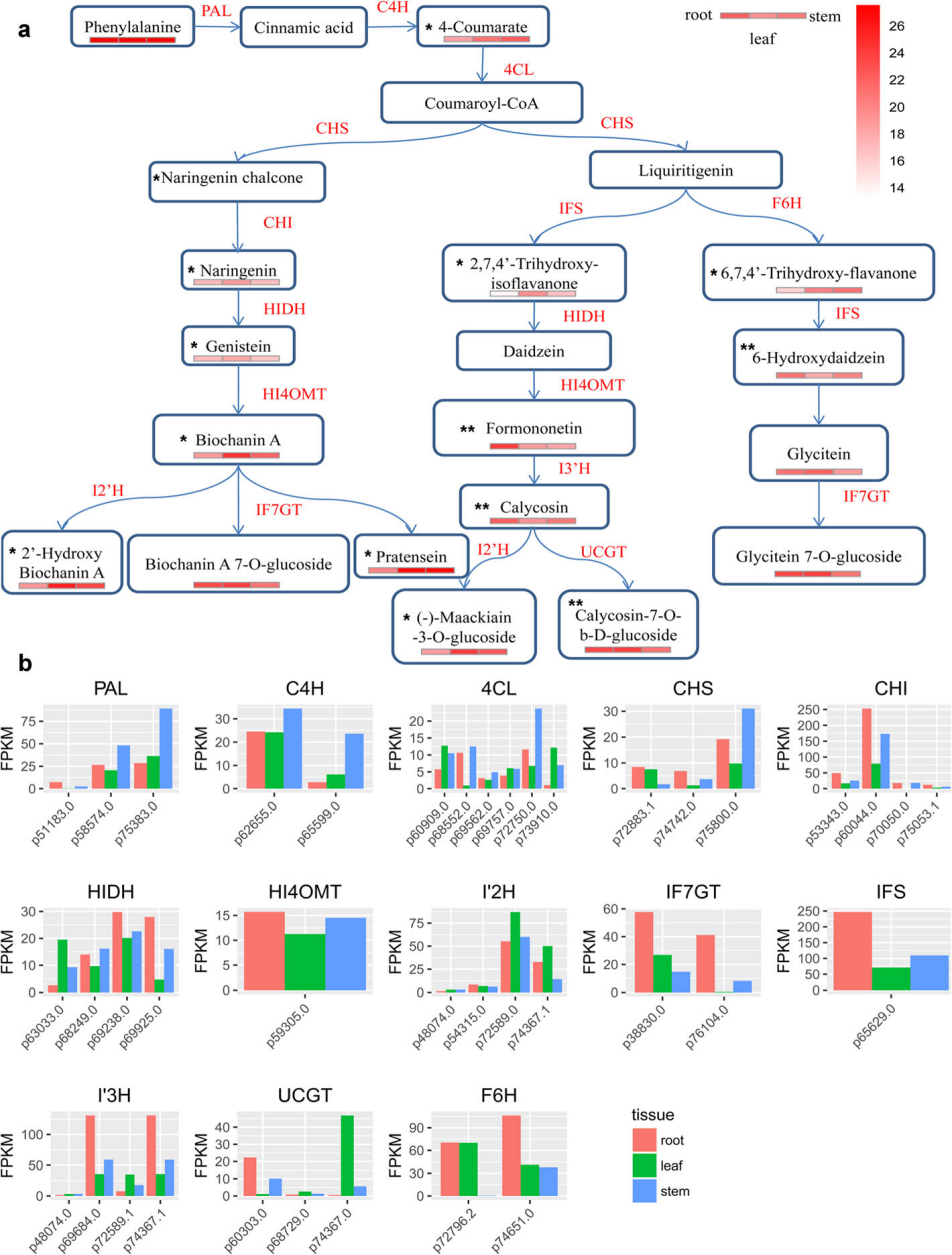
The inferred pathways for phenylpropanoid biosynthesis, flavonoids biosynthesis, and isoflavonoids biosynthesis in AMM. **a** The phenylpropanoid,flavonoids and isoflavonoids biosynthesis pathway. The relative contents (log2GroupArea) for each metabolite in the three tissues were denoted with juxtaposed colored boxes (left: root; middle: leaf; and right: stem). The metabolites that significantly higher in root than the other two tissues were marked by ‘**’, while the metabolites that significantly lower in root than the other two tissues were marked by ‘*’. The enzymes confirmed by transcriptomics data are shown in red. PAL, phenylalanine ammonia-lyase; C4H, cinnamic acid 4-hydroxylase; 4CL, 4-coumarate CoA ligase; CHS, chalcone synthase; CHI, chalconeisomerase; CHR, chalconereductase; IFS, isoflavone synthase; IOMT, Isoflavone O-methyltransferase; I3’H, isoflavone 3′-hydroxylase; I2’H, isoflavone 2′-hydroxylase; UCGT, under calycosin 7-O-glucosyltransferase [2]; HIDH,2-hydroxyisoflavanone dehydratase; HI4OMT,isoflavone 4′-O-methyltransferase; IF7GT, isoflavone 7-O-glucosyltransferase . **b** The expression of the genes (enzymes) in the pathway measured by FPKM value

Although some of the flavonoids were sporadically reported for AMM, we sought to assay and present the complete distribution of flavonoids synthesis in different AMM tissues. Flavonoids are synthesized from condensation of phenylpropanoid derivatives with malonyl-CoA. By integrating the metabolomic and the transcriptomic data, we inferred the complete pathways for flavonoids biosynthesis in AMM, and filled in many gaps in previous works. Downstream of Coumaroyl-CoA production, we for the first time, found all three branches of isoflavonoids biosynthesis in AMM, completing the picture of biosynthesis of Calycosin, Biochanin A, and Glycitein in AMM (Fig. 4a). The latter two were reported for the first time in AMM.

First, in the ‘Calycosin’ branch that we defined previously in AMM [2], in addition to CG, we identified (−)-Maackiain-3-O-glucoside (also named Trifolirhizin) that was not reported in AMM before. It confirmed the activity of I’2H predicted in AMM. Second, the ‘Biochanin A’ branch that we newly defined in AMM, started with Naringenin chalcone, and completed with Biochanin A and its various derivatives. Isoflavone 7-O-glucosyltransferase (IF7GT), the critical enzyme in the pathway, was first identified for AMM by our current study (Fig. 4a). The relative contents of metabolites in this branch were mostly higher in leaf than in other two tissues. Third, the ‘Glycitein’ branch started with liquiritigenin and completed with Glycitein 7-O-glucoside (also known as glycitin). The pathway was completed characterized in AMM with metabolite contents defined for all tissues and newly identified F6H and IF7GT for AMM. We found the relative intensities of metabolites were, in general, consistent with the expression level of enzymes (Fig. 4b).

However, we observed more complex situations, like I3’H and UCGT that synthesize Calycosin and CG. They have many isoforms with different expression patterns in AMM tissues. Two isoforms of I3’H, p69684.0 and p74367.1, and one isoform of UCGT, p60303.0, were highly expressed in root, whereas other I3’H isoforms, p48074.0 and p72589.1, and UCGT isoforms, p74367.0 and p68729.0, were highly expressed in leaf. They might contribute to the synthesis of Calycosin and CG differently in root or leaf due to unequal enzymatic activities. Another likely explanation for the relative higher intensity of Calycosin and CG in root is because of the transportation and storage mechanism in root [65].

### Identification of novel flavonoid derivatives and their inferred pathways

**Fig. 5:**
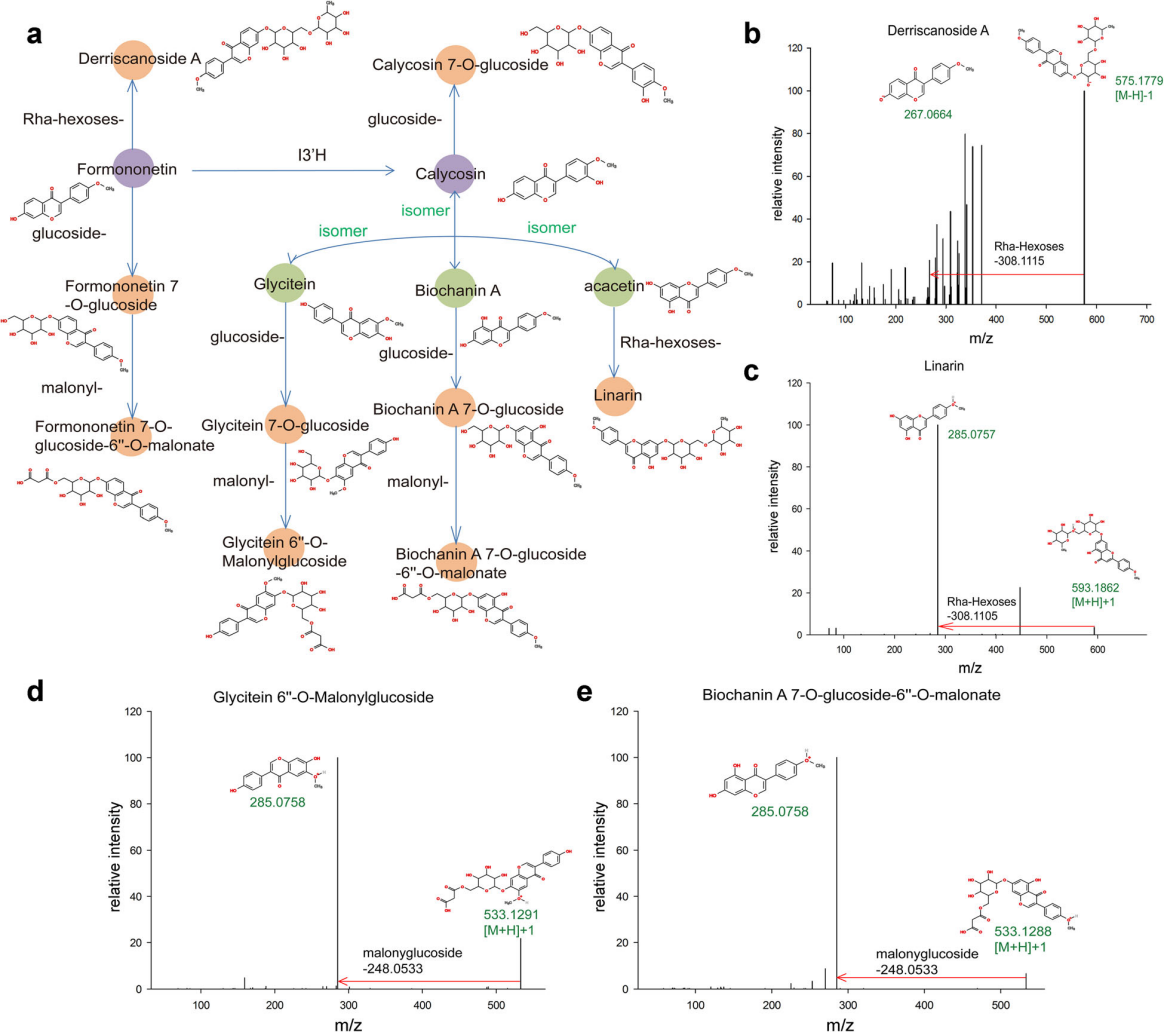
Derivatives of isoflavonoids for some bioactive components from AMM. **a** Derivatives of isoflavonoids of the modifications by glycoside-, malonyl-, and Rha-hexoses- groups are illustrated. **b-e** Mass spectra and structures of novel derivatives from modifications with malonylglucoside, or Rha-hexoses groups in AMM. **b** Derriscanoside A (m/z: 575.1779) is a derivative of formononetin (m/z: 267.0664) with modification of a Rha-hexose-group. **c** Linarin (m/z: 593.1862) is a derivative of acacetin (m/z: 285.0757) with modification of a Rha-hexose-group. **d** Glycitein 6″-O-Malonylglucoside (m/z: 533.1291) is a derivative of glycitein with malonylglucoside modification. **e** Biochanin A 7-O-glucoside-6″-O-malonate (m/z: (533.1288) is a derivative of Biochanin A with malonylglucoside modification

Biochanin A was glycosylated to produce Biochanin A 7-O -D-glucoside (also known as Sissotrin) (Fig. 5). Glycitein was subjected to similar modifications in AMM, generating Glycitein 7-O-glucoside (Fig. 5a). Interestingly, Calycosin not only underwent glycosylation to form (−)-Maackiain-3-O-glucoside (also known as Trifolirhizin) and Calycosin 7-O-glucoside, its precursor Formononetin, and isomer Acacetin were found to undergo novel modifications with Rha-hexose- group to form novel derivatives, Derriscanoside A and Linarin, first identified by our MS data (Fig. 5b, c). In addition, glycosylated flavonoids can be further acylated, such as the novel modifications with malonyl- group, leading to production of Formononetin 7-O-glucoside-6″-O-malonate, Glycitein 7-O-glucoside − 6″-O-Malonylglucoside, and Biochanin A 7-O-glucoside-6″-O-malonate (Fig. 5d, e). Our work reveals the formation of complex derivatives in AMM that were synthesized by extending the isoflavonoids biosynthesis pathway, in which some are first reported by current study.

**Fig. 6:**
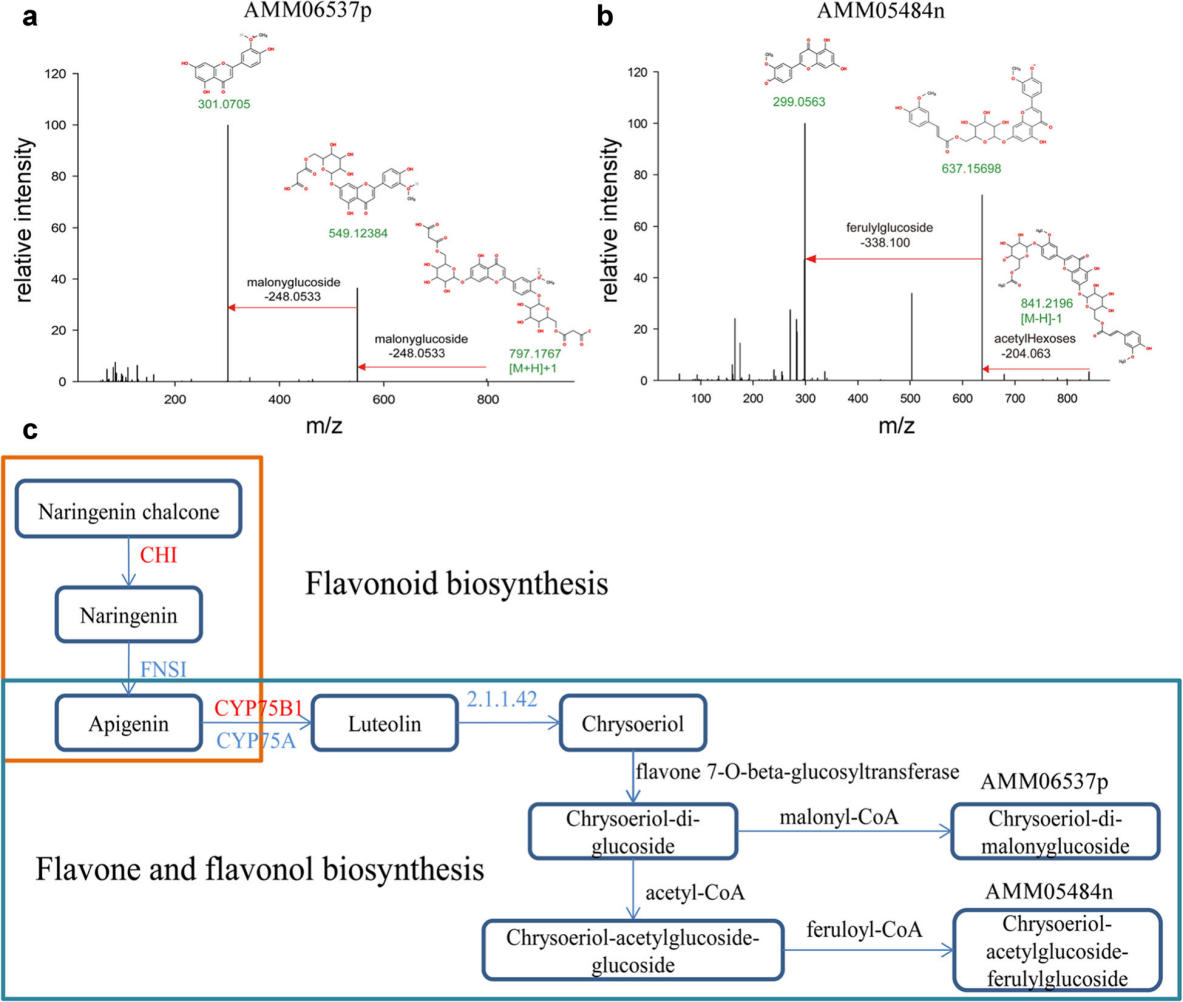
Mass spectra and structures of novel derivatives of Chrysoeriol. **a** AMM06537p (m/z 797.1767) is a derivative of Chrysoeriol (m/z [M + H]+: 301.0705) with modification of two malonylglucoside group. **b** AMM05484n (m/z 841.2196) a derivative of Chrysoeriol (m/z [M-H]-: 299.0563 with modification of an acetyl-hexose-group. **c** The inferredbiosynthetic pathway of the novel derivatives of Chrysoeriol. CHI, chalconeisomerase; FNSI, flavone synthase I

Flavonoids are a class of metabolites that have a core diphenylpropane backbone (C6-C3-C6) (aglycone) with various modifications. In our bioinformatics pipeline [34], we explored the pattern for the fragmentation of flavonoids based on the backbone in order to detect other novel flavonoids with similar MS2 signature of known flavonoids. We documented a number of signature MS2 profiles for flavonoids, including Calycosin, Formononetin, Kaempferol, Apigenin, Chrysoeriol, etc. In addition, we collected a list of neutral losses on flavonoids, which including hexoses, pentoses, malonyl, acetyl, etc. We searched for the presence of above precursor ions and neutral loss in the MS2 spectra of unknown metabolites. As a result, many novel flavonoid derivatives were discovered in our study. One example, AMM06537p (Chrysoeriol-di-malonylglucoside) was detected at RT 6.236 min, and, had the precursor ion at m/z 797.1767 ([M + H]+). Its MS2 spectra displayed the fragment ions of Chrysoeriol that was observed at m/z 301.0705([M + H]+) due to the losses of the two -malonylglucoside groups (Fig. 6a). Another example, AMM05484n (Chrysoeriol-acetylglucoside-ferulylglucoside) was detected at RT 7.489 min, and had the precursor ion at m/z 841.2196 ([M-H]-). Its MS2 spectra displayed the fragment ions at m/z 637.15698 (the neutral loss of a -acetyHexoses group from precursor), and at m/z 299.0563 (fragment ion of Chrysoeriol ([M-H]-), the neutral loss of a -ferulylglucoside group from precursor) (Fig. 6b). Chrysoeriol is a dietary methoxyflavonoid with antioxidant, anti-inflammatory, lipase inhibitory, anti-cancer activities [66–68]. While the synthesis pathways of the novel Chrysoeriol derivatives was unknown, we inferred their likely synthesis steps based on their modification groups, i.e. malonylglucoside, acetylglucoside and ferulylglucoside (Fig. 6c). Accordingly, AMM06537p synthesis can be accomplished by consecutive actions of flavone 7-O-beta-glucosyltransferase and malonyl-CoA. For AMM05484n synthesis, the consecutive actions of flavone 7-O-beta-glucosyltransferase, acetyl-CoA, and feruloyl-CoA are required (Fig. 6c). Taken together, we demonstrated our integrated metabolomics and transcriptomics approach offers great potentials in discovering novel metabolite structure and associated synthesis pathways, which helps provide a comprehensive insight into the complex metabolic network in AMM.

## Conclusions

An integrated metabolomics and transcriptomis analysis approach was taken to study the metabolic profile and synthesis of bioactive compounds in traditional herb *A. membranaceus Bge. var. mongolicus (Bge.) Hsiao*. An unprecedented number of metabolites from AMM was discovered and annotated, representing an increase by an order of magnitude over previous studies. Metabolic profiling found the contents of the main bioactive ingredients were significantly enriched in root, whereas contents and synthesis activity of other phytochemicals were significantly higher in leaf and stem. Using integrated metabolomics and transcriptomics data, we illustrated the complete pathways of phenylpropanoid biosynthesis, flavonoids biosynthesis, and isoflavonoids biosynthesis, for which some are first reported in AMM. More importantly, by combing metabolomics and transcriptomics analysis, we discovered novel flavonoids derivatives, and inferred likely synthesis mechanism. The current study represents the most comprehensive metabolomics analysis on traditional herb AMM, and provides novel insights into the diversity and biosynthesis of metabolites in herbs in general.

## Methods

### Plant materials

AMM were grown for 100 days at 22 °C under a 14 h light/10 h dark photoperiod regime in pots that contained a mixture of vermiculite, black soil and perlite (9:3:0.5). The plants were irrigated with water and fertilized once a week. The 100-day-old plants were harvested, and separated into root, stem and leaf tissues before being frozen immediately in liquid nitrogen and stored at − 80 °C until experimentation [2, 69].

### RNA isolation and transcriptome sequencing

Total RNA was extracted from AMM tissues with RNA prep Pure Plant Kit (Tiangen Biotech, Beijing).. Transcriptome sequencing was performed using BGISEQ-500 at BGI (Shenzheng, China) according to the manufacturer’s protocol. Briefly, RNA-seq library construction started with enrichment of mRNA with polyA tail using Oligo (dT) magnetic beads. Then mRNA was fragmented and used to synthesize double-strand cDNA (dscDNA) by reverse transcription with N6 random primer. dscDNA was end- repaired with phosphate at 5′ end and stickiness ‘A’ at 3′ end, before ligated with adaptor with stickiness ‘T’ at 3′ end. The ligation products were amplified by PCR using two specific primers before they were heat-denatured. The single-strand DNA was cyclized by splint oligo and DNA ligase. The cDNA library was sequenced on a BGISEQ-500 machine to generate 50 bp single-end reads.

### Sequence data mapping and transcriptome analysis

Raw sequencing data were first cleaned by removing reads with adaptor sequences, with more than 10% ‘N’ bases, or with over 50% low quality bases (with quality value < 5). Bowtie2 [70] (version 2.1.0) were used to align clean reads to the reference unigenes, and HISAT [71] were used to align clean reads to the AMM reference transcriptome [2]). Default parameters were used except the SE reads parameter was chosen when it was appropriate.

Gene expression abundance was estimated using the FPKM (Fragments Per Kilobase of exon model per Million mapped reads) value of reads that were mapped to each gene. The calculation of FPKM for each gene was performed with RSEM, which computes Maximum likelihood abundance estimates using the Expectation Maximization (EM) algorithm for its statistical model [72]. NOISeq was used to analyze the differentially expressed genes (DEGs) among different tissues (Fold change ≥2 and diverge probability ≥0.8) [73]. DEGs were further analyzed by gene ontology (GO) enrichment using GOseq, and Kyoto Encyclopedia of Genes and Genomes (KEGG) enrichment using KOBAS software.

### Sample preparation and LC-MS/MS analysis

For AMM tissues, root, stem or leaf (three biological replicates samples for each tissue), 150 mg of sample was ground into powder in liquid nitrogen, followed by extraction with 1 mL 70% (v/v) aqueous methanol solution in an ultrasonic-assisted extraction machine. The sample was reversely blended every 10 min for 3 times before standing at 4 °C for 24 h. The supernatant was transferred into a new 5 ml centrifuge tube after centrifuging at 12,000 g for 10 min at 4 °C. The extraction was repeated for two more times. All the supernatant of the same sample was pooled into the same tube and subsequently treated with a Nitrogen blowing apparatus to volatilize methanol before vacuum dried. Dried extract was re-suspended in 150 μL 70% aqueous methanol solution (containing 1 mg L^− 1^ capsaicin and lincomycin as internal standard). The sample extract was filtered using a 0.22 μm filters (ANPEL, Shanghai) and transferred into a LC glass vial with fused glass insert for analyses.

Chromatographic separation was performed on a Waters Acquity Ultra Performance LC (UPLC) using an ACQUITY UPLC BEH C18 column (pore size 1.7 μm, length, 2.1*100 mm). The mobile phase consisted of (A) water and (B) acetonitrile with 0.04% acetic acid respectively. The following gradient was used for separation with flow rate at 0.25 mL min^− 1^: 95:5 A/B at 0 min, 5:95 A/B at 20.0 min, 5:95 A/B at 24.0 min, 95:5 A/B at 24.1 min, 95:5 A/B at 30.0 min. The sample injection volume was 5 μL, and column temperature was maintained at 40 °C.

Non-targeted metabolomics analysis was performed on Q-Exactive™ Hybrid Quadrupole-Orbitrap High Resolution Mass Spectrometer (Thermo Fisher Scientific) coupled to the UPLC system. Three biological replicates for each AMM tissue were analyzed, which were subjected to the same LC-Q-Orbitrap-MS system and operated under identical instrument conditions. MS/MS-acquisition was performed in both positive and negative ionization in Full MS/dd-MS2 mode, in which the MS2 data of the most abundant ions could be automatically obtained. Heated electrospray ionization (HESI) parameters were as follows: Spray voltage (+), 4000 V; Spray voltage (−), 3500 V; Capillary temperature, 320 °C; Sheath gas, 35 arb; Aux gas, 8 arb; Max spray current, 100 mA; Probe heater temperature, 350 °C; S-Lens RF level, 50. MS full scan mass resolution was set to 70,000 at m/z 200, and the scan range was 100–1000 m/z. Normalized HCD energies were 15 eV and 40 eV, and average MS/MS spectrum was retained. Blank control samples were used periodically to monitor the stability of the analytical conditions.

### Integrated informatics pipeline for MS data processing and identification of metabolites

An informatics pipeline [34] for MS data analysis was created by integrating the following tools/libraries. Compound Discovery software (CD v2.0; Thermo Fisher Scientific) was used to align MS data using its automatic untargeted metabolomics workflow. The following parameters were used: Min Peak Intensity: 5*10E5; Signal/Noise ratio (S/N) Threshold: 10; Mass Tolerance: 5 ppm; Max shift: 1 min; RT Tolerance: 0.1 min. For the others default parameters were used. All the spectra signals were grouped to merged features for positive and negative mode, respectively. After removing redundancy from multi-ion adducts and isotopes, in-source fragmentation, and dimerization, metabolite features with [H] + and [H]- were retained for the positive and negative mode respectively. The GroupArea that assigned by the Compound Discovery software was used to represent the relative content of the metabolites, which displays the median chromatographic peak area for the compound in the sample group.

Raw metabolite features were processed by removing redundancy from multi-ion adducts and isotopes, in-source fragmentation, and dimerization. Then MS signals were also filtered with Group Coefficient of variation (Group CV) < 50%, and Group area > 1E5, to remove artificial signals that have poor quality or non-biological origin. The fragmentation patterns of the remaining high quality metabolite features were extracted using Xcalibur software (v2.2.0). Then metabolite features from positive and negative mode ([H] + mode or [H]-) were merged to eliminate redundancy using parameters: mass value (error < 5 ppm) and retention time (error < 0.5 min).

Metabolites were annotated first using the experimental spectral data from public databases (m/z tolerance < 10 ppm): MassBank [74](http://www.massbank.jp/) and METLIN [75](http://metlin.scripps.edu/index.php). Compounds with similarity score > 0.8 were retained for annotation of the query features. Those that had no match were then annotated using MetFrag with virtual reference compound databases: KEGG and Bio-pubchem [28, 76]. Bio-pubchem was a subset of pubchem, that contained 35,954 compounds of biological origin. MetFrag tool (v2.3) [76] was downloaded from http://c-ruttkies.github.io/MetFrag/ and was used to generate in silico MS/MS spectra of compounds from in KEGG or Bio-pubchem databases and calculate the similarity score between the query MS and reference compounds’ MS. For a query MS, a ranked list of reference compounds was generated (mass error < 5 ppm). The compound classification was carried out based on KEGG Phytochemical compounds library (br08003.keg).

### Statistical data analysis

Principal Component Analysis (PCA) and orthogonal partial least squares discriminate analysis (OPLS-DA) was carried out using SIMCA-P (version 14). Metabolites with significant differences in content were determined with threshold of VIP (variable importance in the projection) > 1 and fold change ≥2 or ≤ 0.5. Other data analysis carried out by in-house scripts were almost performed with R or Perl, such as removing redundancy, assigning the annotations to the metabolites, and some statistical tests. The figures in the manuscripts were almost drawn by R.

## Supplementary information

**Additional file 1.** The workflow of the experiment and bioinformatics pipeline of AMM.

**Additional file 2.** The metabolites from AMM, containing 5435 metabolites with 3985 tagged with MS2 data.

**Additional file 3.** The metabolites from AMM with annotation.

**Additional file 4.** Metabolites previously identified in AMM that are included in our study.

**Additional file 5.** Mapped AMM metabolites in pathways for biosynthesis of secondary metabolites.

**Additional file 6.** Unigenes differentially expressed in leaf and stem in comparison of root transcriptome.

**Additional file 7.** The differential metabolites and genes in different tissues of AMM.

**Additional file 8.** The distribution of metabolites and the PCA and OPLS-DA analysis of metabolites in AMM tissues.

**Additional file 9.** OPLS-DA analysis of metabolites between stem and root.

**Additional file 10.** The distribution of the relative content (log2GroupArea) for different metabolite groups.

## Data Availability

The datasets analyzed during the current study available from the corresponding author on reasonable request.

## Abbreviations

AMM: *Astragalus membranaceus Bge. var. mongolicus (Bge.) Hsiao*
CG: Calycosin-7-O-b-D-glucoside
ASI: Astragaloside IV
MS: Mass spectrum
LC-MS: Liquid chromatography-mass spectrum
MS2: MS-MS, the secondary mass spectrum
PCA: Principal Component Analysis
OPLS-DA: Orthogonal partial least squares discriminate analysis
VIP: Variable importance in the projection
CAS: Cycloartenol synthase
P450: CytochromeP450
GT: Glycosyl transferase
IF7GT: Isoflavone 7-O-glucosyltransferase
