# Supplementary figures and images for: Integrated metabolomics and transcriptomics study of traditional herb Astragalus membranaceus Bge. var. mongolicus (Bge.) Hsiao reveals global metabolic profile and novel phytochemical ingredients
Source: BMC Genomics. 2020 Nov 18;21(Suppl 10):697. doi: 10.1186/s12864-020-07005-y (PMC7677826; doi:10.1186/s12864-020-07005-y)

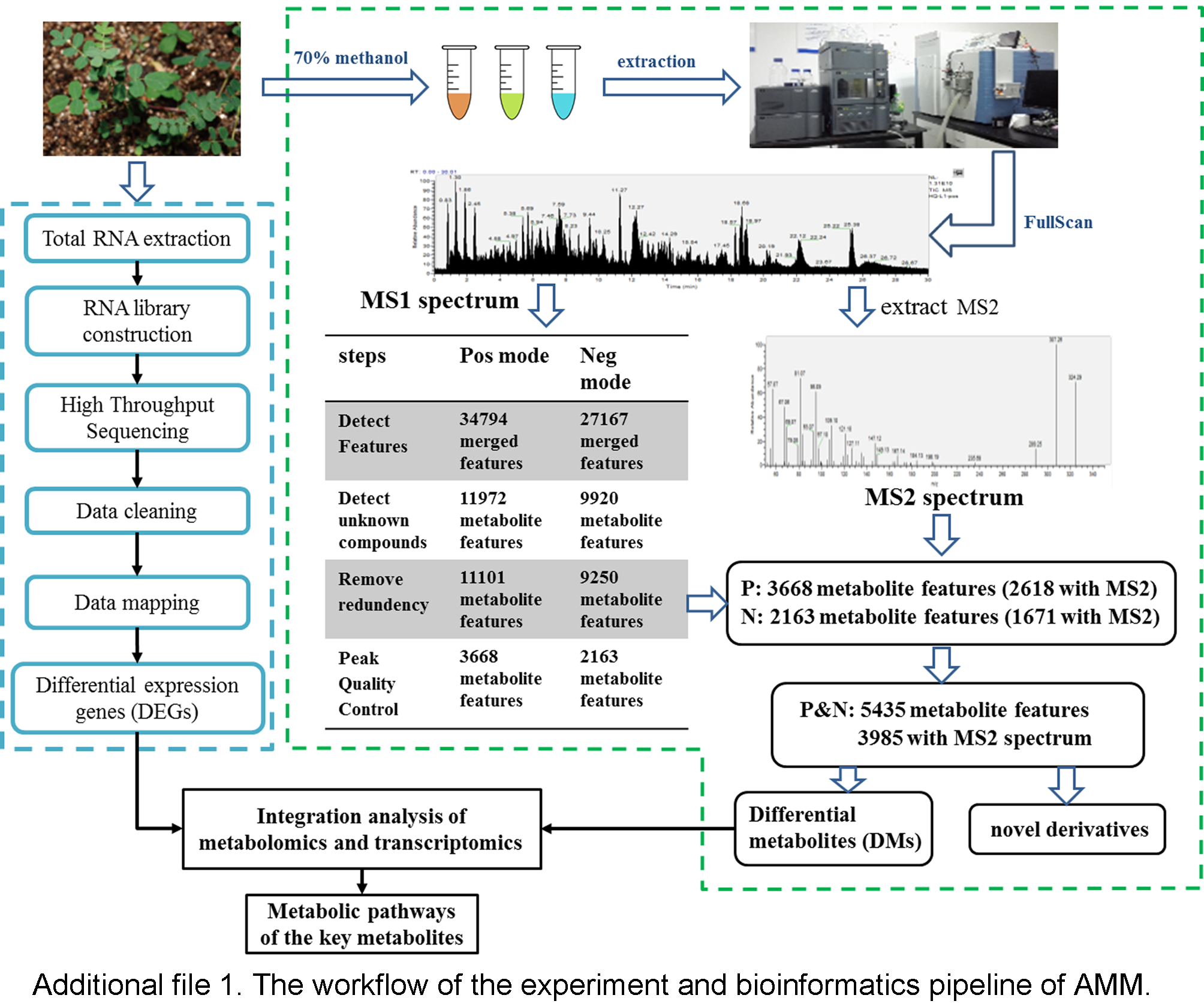

Supplement: Supplementary file 1 — Additional file 1. The workflow of the experiment and bioinformatics pipeline of AMM. [file 12864_2020_7005_MOESM1_ESM.tif]

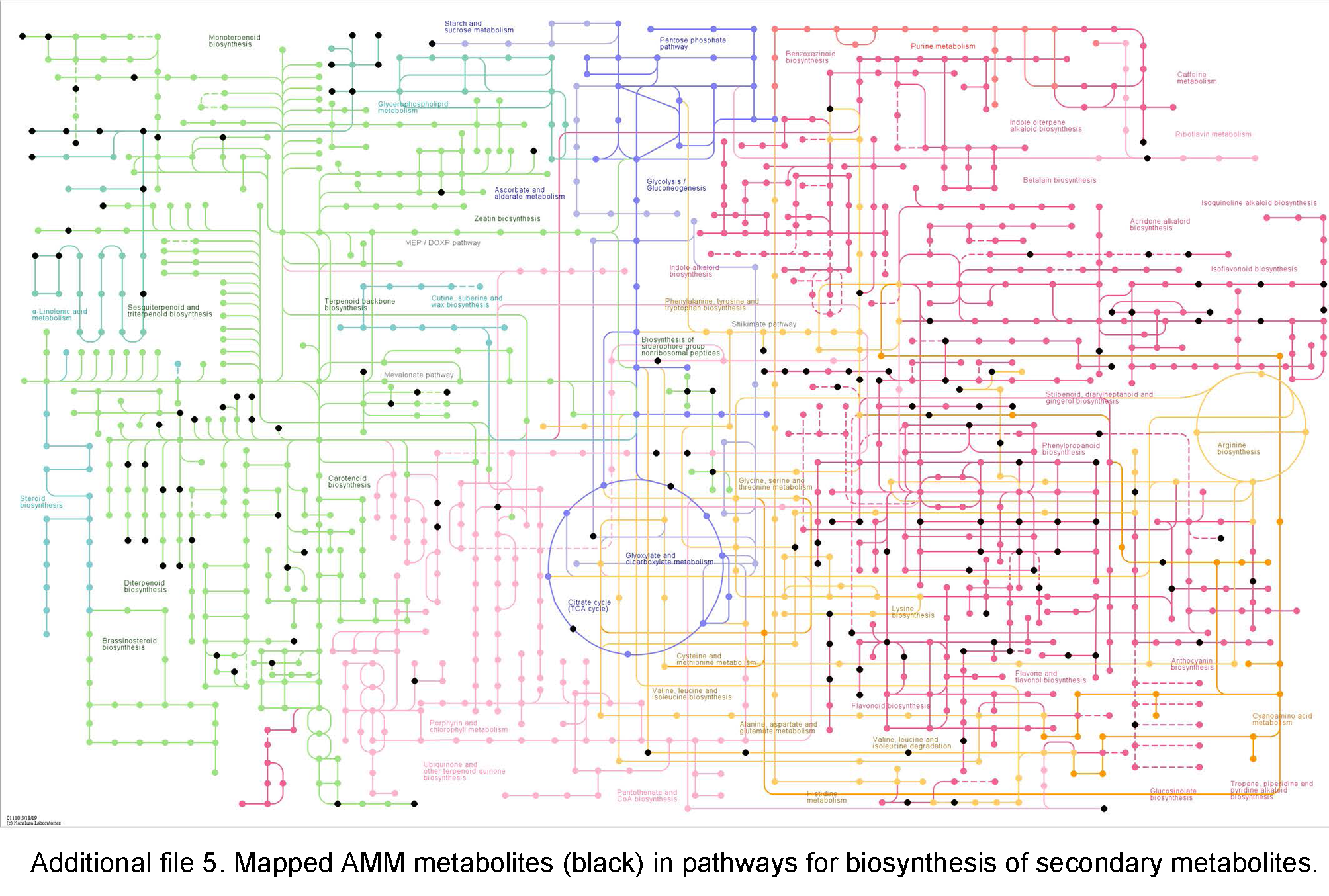

Supplement: Supplementary file 5 — Additional file 5. Mapped AMM metabolites in pathways for biosynthesis of secondary metabolites. [file 12864_2020_7005_MOESM5_ESM.tif]

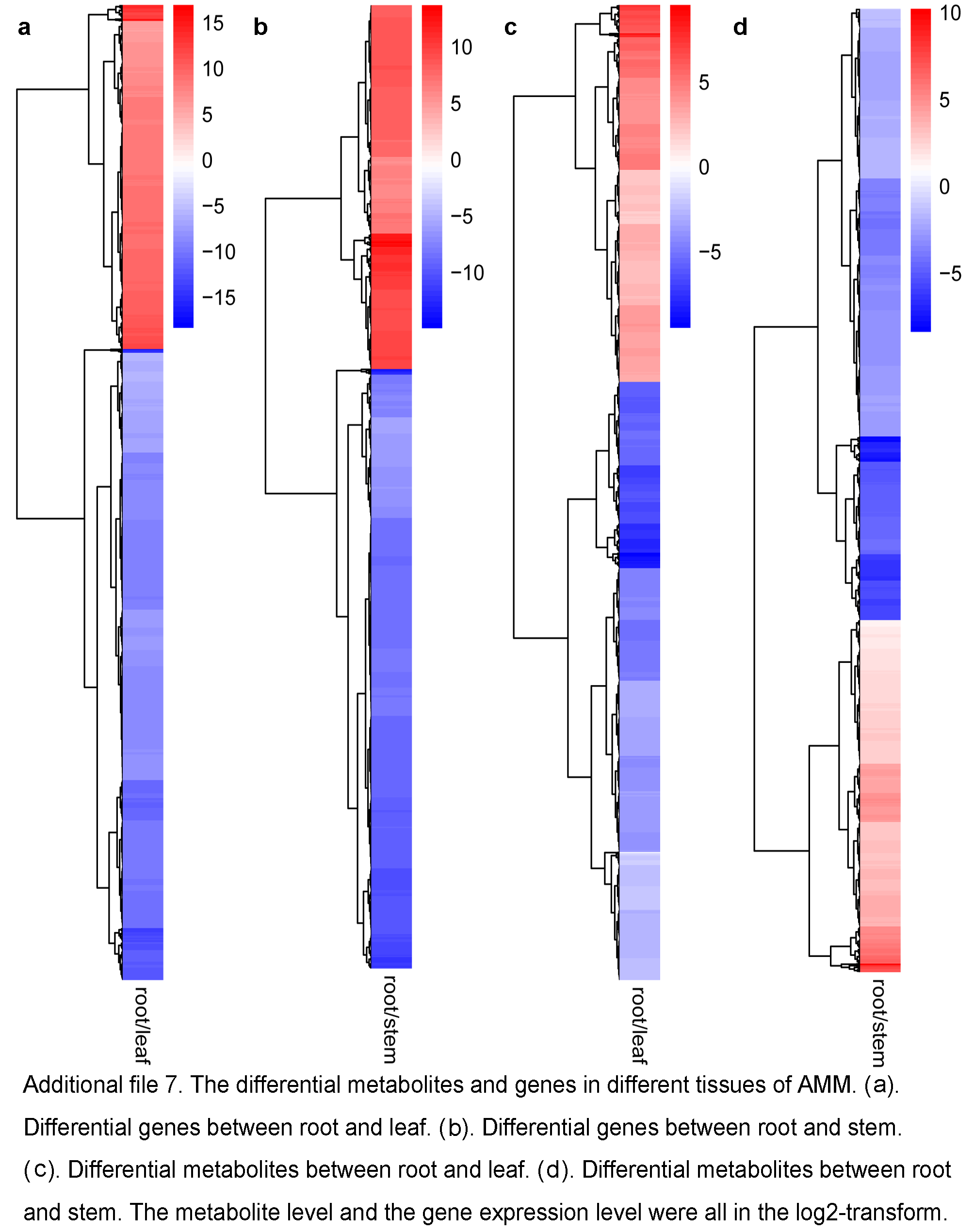

Supplement: Supplementary file 7 — Additional file 7. The differential metabolites and genes in different tissues of AMM. [file 12864_2020_7005_MOESM7_ESM.tif]

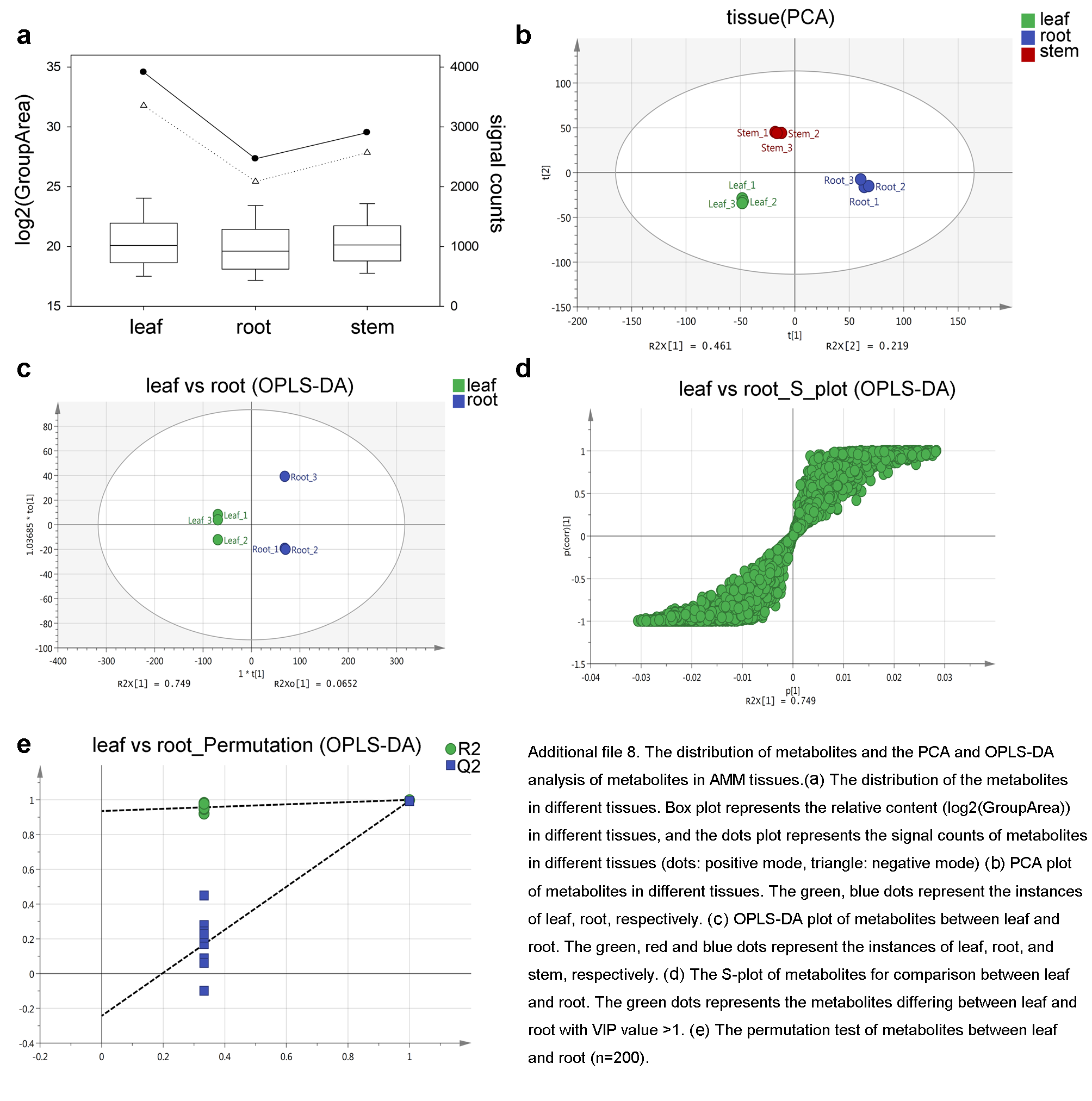

Supplement: Supplementary file 8 — Additional file 8. The distribution of metabolites and the PCA and OPLS-DA analysis of metabolites in AMM tissues. [file 12864_2020_7005_MOESM8_ESM.tif]

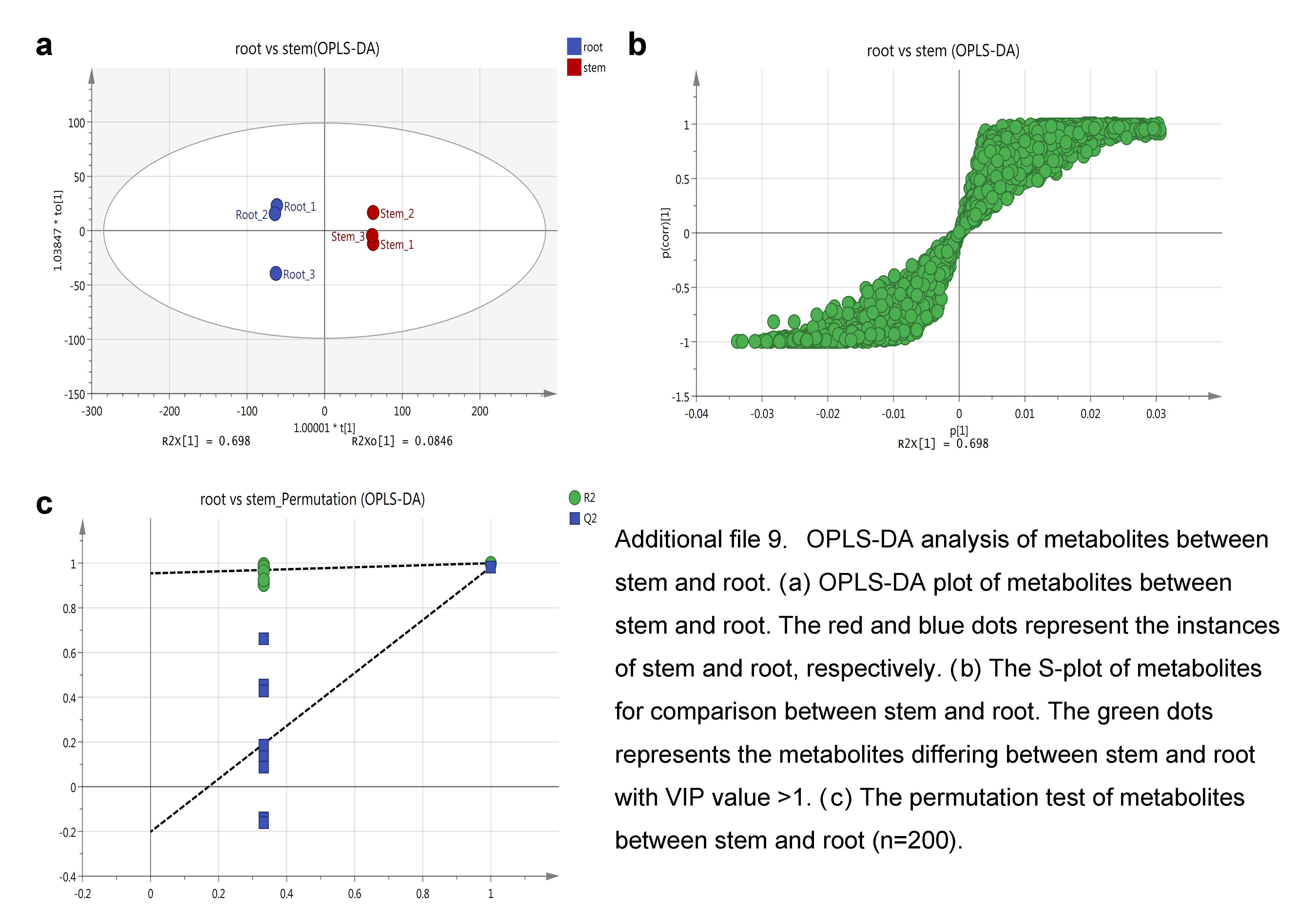

Supplement: Supplementary file 9 — Additional file 9. OPLS-DA analysis of metabolites between stem and root. [file 12864_2020_7005_MOESM9_ESM.tif]

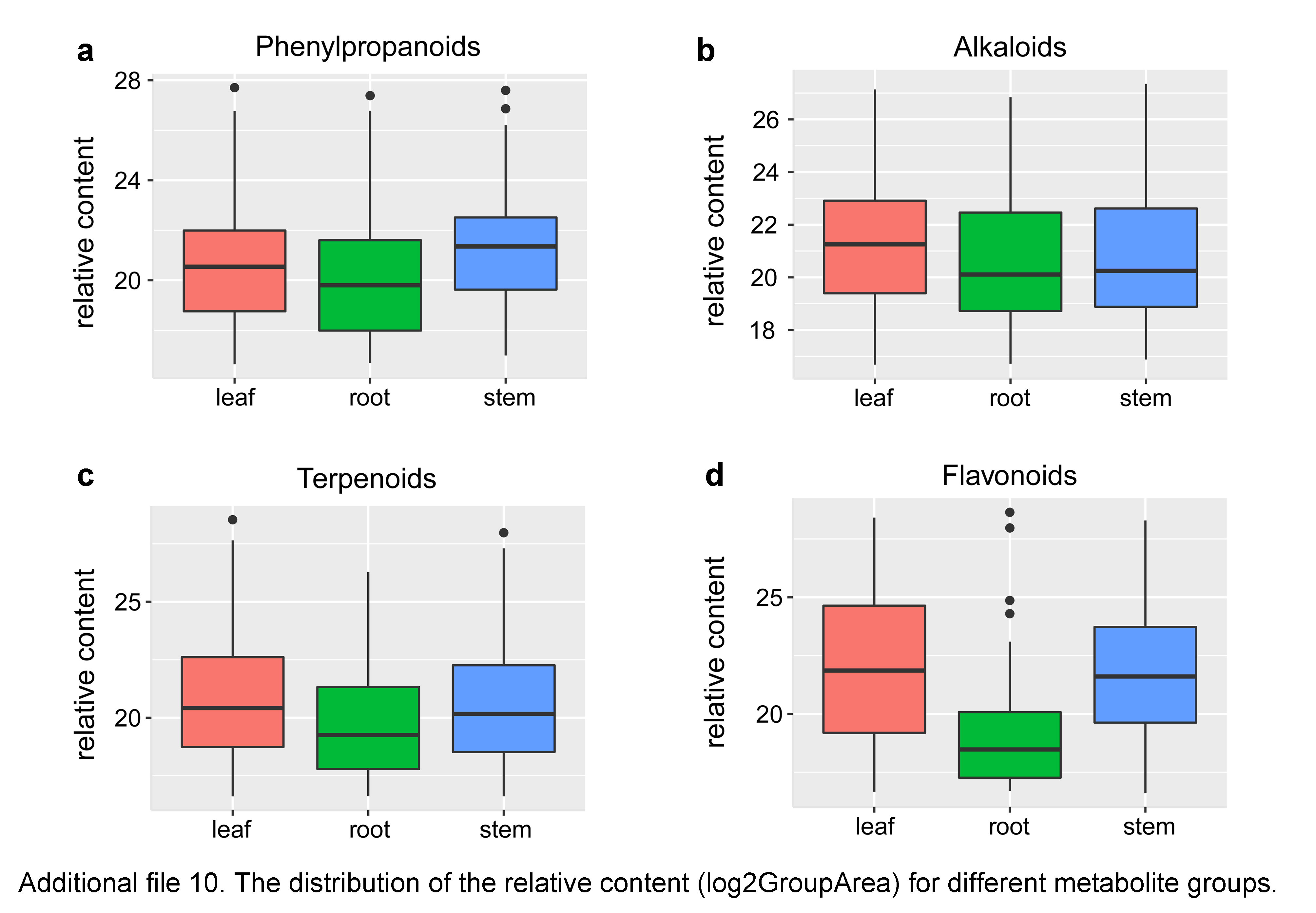

Supplement: Supplementary file 10 — Additional file 10. The distribution of the relative content (log2GroupArea) for different metabolite groups. [file 12864_2020_7005_MOESM10_ESM.tif]
