# Supplementary material for: Integrated metabolomics and transcriptomics study of traditional herb Astragalus membranaceus Bge. var. mongolicus (Bge.) Hsiao reveals global metabolic profile and novel phytochemical ingredients
Source: BMC Genomics. 2020 Nov 18;21(Suppl 10):697. doi: 10.1186/s12864-020-07005-y (PMC7677826; doi:10.1186/s12864-020-07005-y)
Supplement: Supplementary file 4 — Additional file 4. Metabolites previously identified in AMM that are included in our study. [file 12864_2020_7005_MOESM4_ESM.doc]

Additional file 4. Metabolites previously identified in AMM that are included in our study.

| Putativename | PutativeFormula | Molecular Weight | Literature |
| --- | --- | --- | --- |
| Astragaloside IV | C41H68O14 | 784.4614 |  |
| Isoastragaloside II | C43H70O15 | 826.4706 |  |
| Astragaloside I | C45H72O16 | 868.4827 |  |
| Soyasaponin II | C47H76O17 | 912.509 |  |
| Astragaloside III | C41H68O14 | 784.4614 |  |
| Astragaloside II | C43H70O15 | 826.4706 |  |
| Isoastragaloside I | C45H72O16 | 868.4827 |  |
| Biochanin A | C16H12O5 | 284.0681 |  |
| Formononetin | C16H12O4 | 268.0737 |  |
| Glycitein | C16H12O5 | 284.0682 |  |
| Formononetin 7-O-glucoside-6''-O-malonate | C25H24O12 | 516.1266 |  |
| Soyasaponin I | C48H78O18 | 942.5176 |  |
| Soyasaponin III | C42H68O14 | 796.4603 |  |
| Soyasapogenol B | C30H50O3 | 458.3766 |  |
| Isorhamnetin | C16H12O7 | 316.058 |  |
| Kaempferol-3-O-glucoside  (Astragalin) | C21H20O11 | 448.1011 |  |
| Palmatine | C21H21NO4 | 351.1469 |  |
| Coumarin | C9H6O2 | 146.0367 |  |
| Trigonelline | C7H7NO2 | 137.0477 |  |
| Proline | C5H9NO2 | 115.0636 |  |
| Leucine | C6H13NO2 | 131.0947 |  |
| Rhamnocitrin | C16H12O6 | 300.0631 |  |
| Calycosin | C16H12O5 | 284.0681 |  |
| Pratensein | C16H12O6 | 300.0631 |  |
| Astrapterocarpan | C17H16O5 | 300.0995 |  |
| Genistein | C15H10O5 | 270.0527 |  |
| Isoquercitrin | C21H20O11 | 448.1011 |  |
| Valine | C5H11NO2 | 117.07922 |  |

**References**

Anderson DM, Howlett JF, McNab CG. 1985. The amino acid composition of the proteinaceous component of gum tragacanth (Asiatic Astragalus spp.). *Food additives and contaminants***2**(4): 231-235.

Chang YX, Ge AH, Donnapee S, Li J, Bai Y, Liu J, He J, Yang X, Song LJ, Zhang BL et al. 2015. The multi-targets integrated fingerprinting for screening anti-diabetic compounds from a Chinese medicine Jinqi Jiangtang Tablet. *Journal of ethnopharmacology***164**: 210-222.

Cui B, Inoue J, Takeshita T, Kinjo J, Nohara T. 1992. Triterpene glycosides from the seeds of Astragalus sinicus L. *Chemical & pharmaceutical bulletin***40**(12): 3330-3333.

Gu Y, Huang ZD, Liu YH. 1997. [Studies on the efficacious constituents of Astragalus complanatus]. *Yao xue xue bao = Acta pharmaceutica Sinica***32**(1): 59-61.

Huang X, Liu Y, Song F, Liu Z, Liu S. 2009. Studies on principal components and antioxidant activity of different Radix Astragali samples using high-performance liquid chromatography/electrospray ionization multiple-stage tandem mass spectrometry. *Talanta***78**(3): 1090-1101.

Jung JY, Jung Y, Kim JS, Ryu DH, Hwang GS. 2013. Assessment of peeling of Astragalus roots using 1H NMR- and UPLC-MS-based metabolite profiling. *Journal of agricultural and food chemistry***61**(43): 10398-10407.

Krasteva I, Platikanov S, Nikolov S, Kaloga M. 2007. Flavonoids from Astragalus hamosus. *Natural product research***21**(5): 392-395.

Kwon HJ, Park YD. 2012. Determination of astragalin and astragaloside content in Radix Astragali using high-performance liquid chromatography coupled with pulsed amperometric detection. *Journal of chromatography A***1232**: 212-217.

Lin LZ, He XG, Lindenmaier M, Nolan G, Yang J, Cleary M, Qiu SX, Cordell GA. 2000. Liquid chromatography-electrospray ionization mass spectrometry study of the flavonoids of the roots of Astragalus mongholicus and A. membranaceus. *Journal of chromatography A***876**(1-2): 87-95.

Lin Q, Li Y, Tan XM, Yao XC. 2013. [Simultaneous determination of formononetin, calycosin and isorhamnetin from Astragalus mongholicus in rat plasma by LC-MS/MS and application to pharmacokinetic study]. *Zhong yao cai = Zhongyaocai = Journal of Chinese medicinal materials***36**(4): 589-593.

Liu EH, Qi LW, Peng YB, Cheng XL, Wu Q, Li P, Li CY. 2009. Rapid separation and identification of 54 major constituents in Buyang Huanwu decoction by ultra-fast HPLC system coupled with DAD-TOF/MS. *Biomedical chromatography : BMC***23**(8): 828-842.

Mitaine-Offer AC, Miyamoto T, Semmar N, Jay M, Lacaille-Dubois MA. 2006. A new oleanane glycoside from the roots of Astragalus caprinus. *Magnetic resonance in chemistry : MRC***44**(7): 713-716.

Ohkawara S, Okuma Y, Uehara T, Yamagishi T, Nomura Y. 2005. Astrapterocarpan isolated from Astragalus membranaceus inhibits proliferation of vascular smooth muscle cells. *European journal of pharmacology***525**(1-3): 41-47.

Qi LW, Cao J, Li P, Yu QT, Wen XD, Wang YX, Li CY, Bao KD, Ge XX, Cheng XL. 2008. Qualitative and quantitative analysis of Radix Astragali products by fast high-performance liquid chromatography-diode array detection coupled with time-of-flight mass spectrometry through dynamic adjustment of fragmentor voltage. *Journal of chromatography A***1203**(1): 27-35.

Qi LW, Li P, Ren MT, Yu QT, Wen XD, Wang YX. 2009. Application of high-performance liquid chromatography-electrospray ionization time-of-flight mass spectrometry for analysis and quality control of Radix Astragali and its preparations. *Journal of chromatography A***1216**(11): 2087-2097.

Roll R, Bar F. 1967. [Effect of coumarin (o-hydroxyeinnamic acid-lactone) on pregnant female mice]. *Arzneimittel-Forschung***17**(1): 97-100.

Shen P, Liu MH, Ng TY, Chan YH, Yong EL. 2006. Differential effects of isoflavones, from Astragalus membranaceus and Pueraria thomsonii, on the activation of PPARalpha, PPARgamma, and adipocyte differentiation in vitro. *The Journal of nutrition***136**(4): 899-905.

Xu Q, Ma X, Liang X. 2007. Determination of astragalosides in the roots of Astragalus spp. using liquid chromatography tandem atmospheric pressure chemical ionization mass spectrometry. *Phytochemical analysis : PCA***18**(5): 419-427.

Zhang YZ, Xu F, Liang J, Tang JS, Shang MY, Wang X, Cai SQ. 2012. [Isoflavonoids from roots of Astragalus membranaceus var. mongholicus]. *Zhongguo Zhong yao za zhi = Zhongguo zhongyao zazhi = China journal of Chinese materia medica***37**(21): 3243-3248.

Zhang ZX, Qi F, Zhou DJ, Liang XY, Zhu LW, Wang PZ. 2006. [Effect of 5-fluorouracil in combination with Astragalus membranaceus on amino acid metabolism in mice model of gastric carcinoma]. *Zhonghua wei chang wai ke za zhi = Chinese journal of gastrointestinal surgery***9**(5): 445-447.
